# *Trachyphloeosomarutiani* sp. nov., a new species from Guangdong, China (Coleoptera, Curculionidae, Entiminae)

**DOI:** 10.3897/BDJ.13.e142838

**Published:** 2025-02-19

**Authors:** Jiang Zhu, Cheng-Bin Wang, Ye Zhen

**Affiliations:** 1 State Environmental Protection Key Laboratory of Urban Ecological Simulation and Protection, South China Institute of Environmental Sciences, MEE, Guangzhou, China State Environmental Protection Key Laboratory of Urban Ecological Simulation and Protection, South China Institute of Environmental Sciences, MEE Guangzhou China; 2 Mianyang Normal University, Engineering Research Center for Forest and Grassland Disaster Prevention and Reduction, Mianyang, China Mianyang Normal University, Engineering Research Center for Forest and Grassland Disaster Prevention and Reduction Mianyang China

**Keywords:** morphology, new taxon, Oriental Realm, taxonomy, Trachyphloeini, *Trachyphloeosoma*, weevil

## Abstract

**Background:**

From August to September 2024, we led a team of Guangzhou No.5 Binjiang Junior High School students to study the community structure of leaf litter beetles in Guangzhou, Guangdong Province, southern China, to prepare for the Youth Innovation Competition hosted by the Guangzhou Education Bureau. In the study, 479 specimens were collected in total, identified as 73 species in 14 families. Amongst all the specimens, some are considered new species, one of which is described here.

The genus *Trachyphloeosoma* Wollaston, 1869 (Coleoptera, Curculionidae, Entiminae, Trachyphloeini) includes six known species occurring in China and the genus is recorded in Guangdong Province for the first time.

**New information:**

A new weevil, *Trachyphloeosomarutiani* sp. nov. (Coleoptera, Curculionidae, Entiminae), is described from Guangzhou City, Guangdong Province, China. Important morphological characters of the new species are illustrated by colour plates.

## Introduction

The genus *Trachyphloeosoma* Wollaston, 1869 belongs to Coleoptera, Curculionidae, Entiminae, Trachyphloeini and members of this tribe are regarded as small wingless terricolous beetles with limited dispersal ability (Ren et al. 2020). Up to now, the genus includes 16 known species from China, Japan, Vietnam and Philippines (Borovec 2014, Morimoto et al. 2015, Ren et al. 2020, Borovec 2021, Borovec and Anderson 2022, Borovec and Anderson 2024). Amongst them, six species were recorded in China, *Trachyphloeosomaroelofsi* Sharp, 1896, *T.honza* Ren, Borovec & Zhang, 2020, *T.jirka* Ren, Borovec & Zhang, 2020, *T.martin* Ren, Borovec & Zhang, 2020, *T.ales* Borovec & Anderson, 2022 and *T.david* Borovec & Anderson, 2022.

*Trachyphloeosoma* species are covered by brownish, earth-like encrustation with nearly invisible, scarce, irregularly angular adherent scales below (Borovec 2009, Ren et al. 2020, Borovec and Anderson 2022). The earth-like encrustation is made of specialised setae contained with earth. This genus is closely related to *Trachyphilus* Faust, 1890, but can be distinguished by the shape of the scrobe and rostrum (Borovec 2009).

## Materials and methods

**Figure 1.**
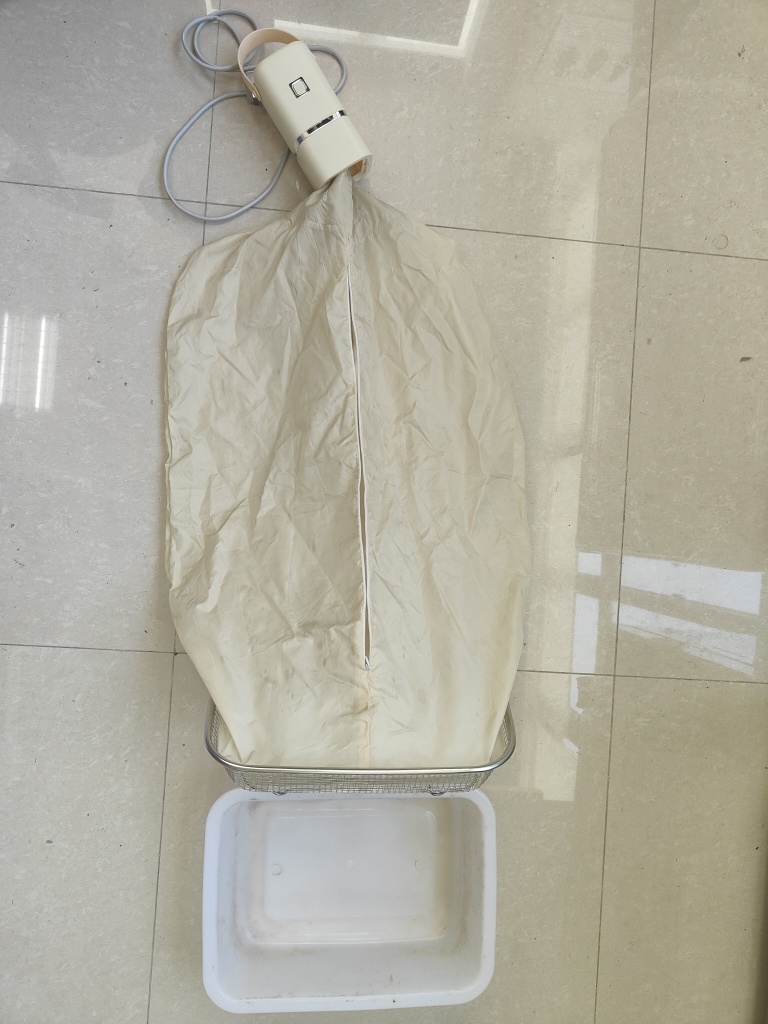
Modified heating dryer.

We set up three gradients with each consisting of three sampling areas, each sampling area consisting of three plots (3 m x 3 m). In each round, a sifter (net diameter 5 mm × 5 mm) was used to separate all the leaf litter in the plot. All sieved residue was stored in breathable cotton bags and carefully labelled with the plot-number. After bringing it back to the State Environmental Protection Key Laboratory, a modified heating dryer was used to separate insects from leaf litter (Fig. 1). A total of two rounds were sampled, with at least two weeks between each round.

Specimens were relaxed and softened in 40℃ hot water for 24 hours, then cleaned by using ultrasonic cleaning machine for 10 mins. Specimens were transferred to distilled water to observe and dissect. In order to examine the genitalia, the abdomens were detached and treated with a 10% solution of potassium hydroxide (KOH) for 12 hours, then transferred to distilled water to flush the remaining KOH and stop any further bleaching. After examination, the body parts were mounted on a glass slide with BASO sealing adhesive (http://www.baso.com.cn/view.asp?id=110) for further studies. All photographs were taken using a Sigma FP-L camera with Mitutoyo M Plan Apo 10X Microscopic lens and two Amaran 200ds lights as light sources. The final deep focus images were created with Helicon Focus 7.7.0 stacking software. Adobe Photoshop CC 2024 was used for post processing.

The holotype is deposited in the Insect Collection of South China Institute of Environmental Sciences (SCIES), Guangzhou, China and the paratypes are deposited in the Insect Collection of Mianyang Normal University (MYNU).

## Taxon treatments

### Trachyphloeosoma rutiani

Zhu, Wang & Zhen sp. nov.

0DCC8A7B-E221-552A-A9AA-9479A5C12565

A7E9A772-FEA2-4DAA-9AB2-FF0839B19B17

#### Materials

- **Type status:** Holotype. **Occurrence:** recordedBy: Jiang Zhu; Rutian Ye; individualCount: 1; sex: female; lifeStage: Adult; occurrenceID: CA0F6CF4-BA6D-5B55-A9AD-BF82125AFDCC; **Location:** country: China; stateProvince: Guangdong; verbatimLocality: Guangzhou City, Tianhe Distrct, near Shaojiwo Reservoir (广东省，广州市，天河区，筲箕窝水库); verbatimElevation: 158 m; decimalLatitude: 23.23465577; decimalLongitude: 113.39828036; geodeticDatum: WGS84; **Event:** verbatimEventDate: 28-09-2024; habitat: leaf litter; **Record Level:** institutionID: SCIES
- **Type status:** Paratype. **Occurrence:** recordedBy: Jiang Zhu; Rutian Ye; individualCount: 1; sex: female; lifeStage: Adult; occurrenceID: 5658556B-4F0A-55C5-90A9-9E1BF034120F; **Location:** country: China; stateProvince: Guangdong; verbatimLocality: Guangzhou City, Tianhe Distrct, near Shaojiwo Reservoir (广东省，广州市，天河区，筲箕窝水库); verbatimElevation: 158 m; decimalLatitude: 23.23465577; decimalLongitude: 113.39828036; geodeticDatum: WGS84; **Event:** verbatimEventDate: 28-09-2024; habitat: leaf litter; **Record Level:** institutionID: MYNU
- **Type status:** Paratype. **Occurrence:** recordedBy: Jiang Zhu, Ru-Tian Ye & Zhen-Yu Piao; individualCount: 1; sex: female; lifeStage: Adult; occurrenceID: 168FC8A7-0BA0-5E0E-A075-D8AE284361EA; **Location:** country: China; stateProvince: Guangdong; verbatimLocality: Heyuan City, Zijin County, Baixi Provincial Nature Reserve; verbatimElevation: 274 m; decimalLatitude: 23.707126; decimalLongitude: 115.175949; geodeticDatum: WGS84; **Event:** verbatimEventDate: 28-09-2024; habitat: leaf litter; **Record Level:** institutionID: MYNU

#### Description

**Figure 2.**
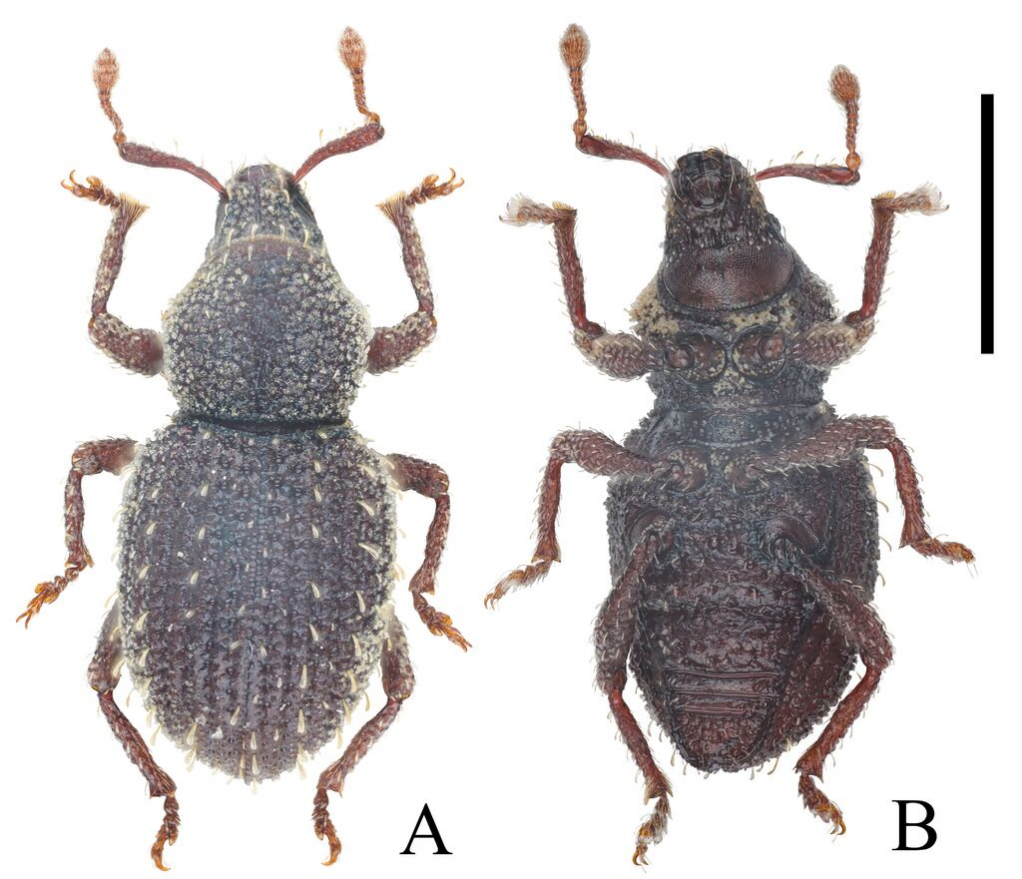
Habitus of *Trachyphloeosomarutiani* sp. nov., female: **A** Dorsal view; **B** Ventral view. Scale Bar = 0.5 mm.

**Holotype female.** Body length: holotype 2.33 mm, paratypes 2.26–2.41 mm. Body (Fig. 2) most rusty dark brown; antennae and legs lighter, reddish-brown. Entire body, except antennal flagellum and tarsi, covered by sparse yellowish scales; antennal flagellum densely covered by short yellowish setae; head, pronotum, elytra except central and lateral areas, legs except tarsi, covered by specialised star-shaped, yellowish, short setae; specialised setae on elytra and legs much finer. Entire body in dermatoglyph texture; head, pronotum and elytra densely coriaceous, all interstriae finely punctate, yellowish erect setae on only odd interstriae.

**Figure 3.**
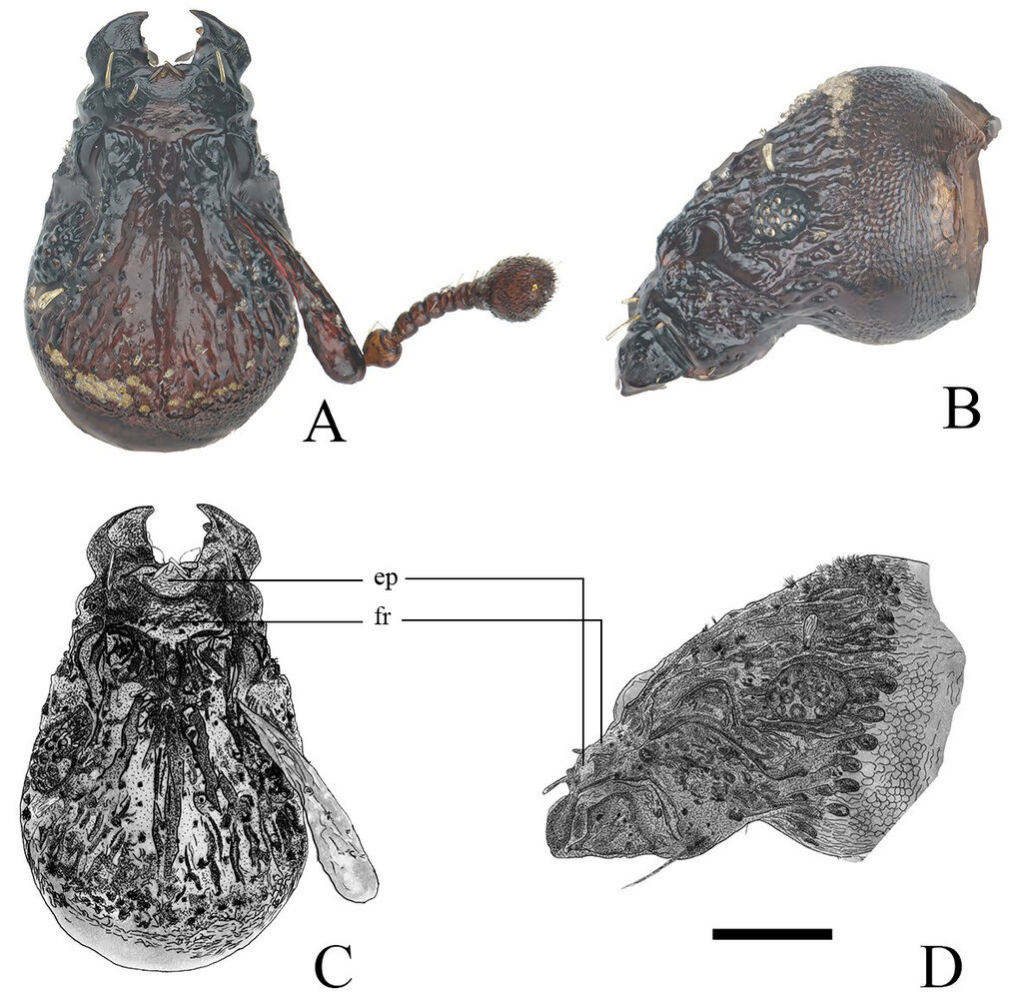
Head of *Trachyphloeosomarutiani* sp. nov., female (setae and microhairs mostly removed). **A, C** Dorsal view; **B, D** Lateral view. Scale Bar = 0.2 mm. Abbreviations: **ep** epistome, **fr** frons.

Head (Fig. 3) coriaceous, finely rugose and punctate, with three main vertical grooves on frons, each groove divided into several sculptures and ridges (Figs 1, 2, 5) on vertex. Rostrum wider than long, tapered anteriad, widest at base. Epistome convex, tilting backwards, separated from frons by crescent-shaped, raised ridge. Scrobes in dorsal view mostly visible, in lateral view short and gradually enlarged posteriad into a separated reniform groove before eyes, opened at posteroventral margin. Rostrum in lateral view slightly vaulted, at the same level as head. Eyes small, convex, oval, slightly prominent from outline of head, composed of about 15 separated facets. Postgena with several waterdrop-shaped grooves in a vertical row, each groove filled with flocculent microhairs.

**Figure 4.**
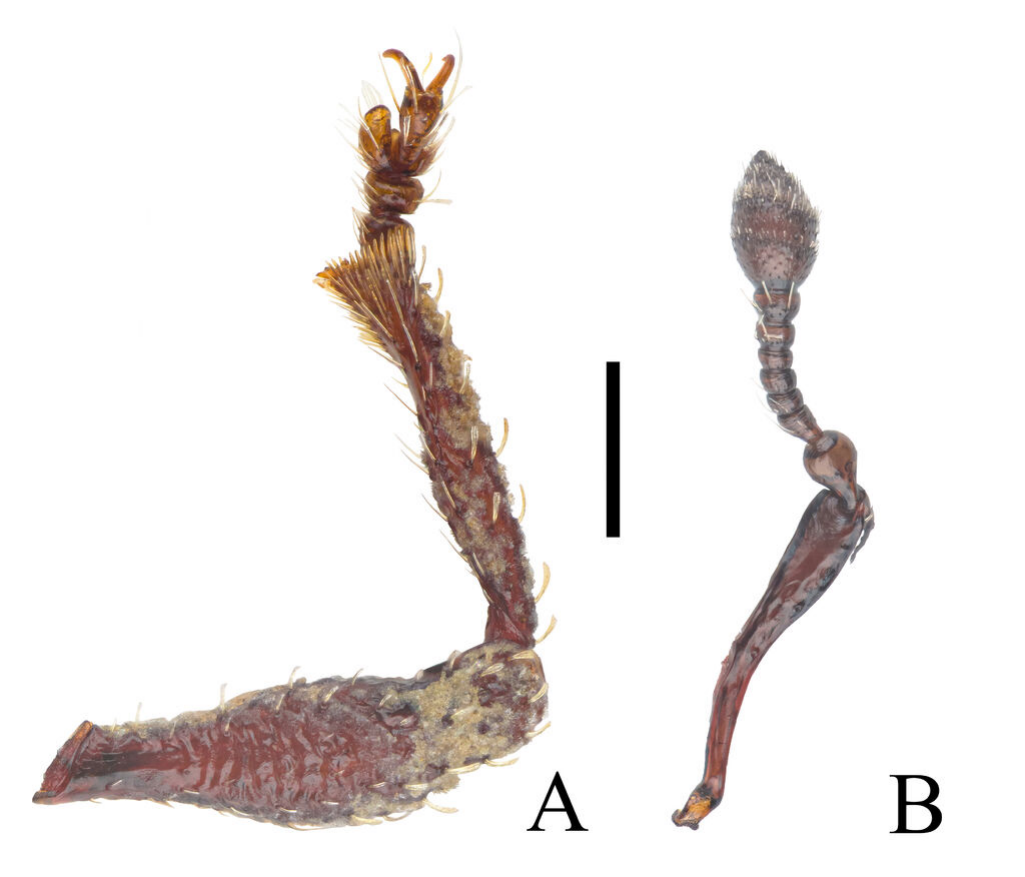
Proleg and antenna of *Trachyphloeosomarutiani* sp. nov., female. **A** Right proleg, anterior view; **B** Right antenna, ventral view. Scale bar = 0.2 mm.

Antennae (Fig. 4B) moderately long. Scape as long as funicle plus club, distinctly curved at proximal half, gradually enlarged apicad, at apex about 0.7× as wide as widest part of club. Funicle with seven antennomeres, antennomeres 1–2 conical, antennomeres 3–4 compressed into short cylinders, antennomeres 5–7 cup-shaped. Club ovoid, large. Length of each antennomere in mm: scape (0.43), funicular antennomere 1 (0.10), funicular antennomere 2 (0.05), funicular antennomere 3 (0.02), funicular antennomere 4 (0.03), funicular antennomere 5 (0.02), funicular antennomere 6 (0.03), funicular antennomere 7 (0.04), club (0.16); width of each antennomere in mm: funicular antennomere 1 (0.06), funicular antennomere 2 (0.04), funicular antennomere 3 (0.04), funicular antennomere 4 (0.04), funicular antennomere 5 (0.04), funicular antennomere 6 (0.05), funicular antennomere 7 (0.06) and club (0.10).

**Figure 5.**
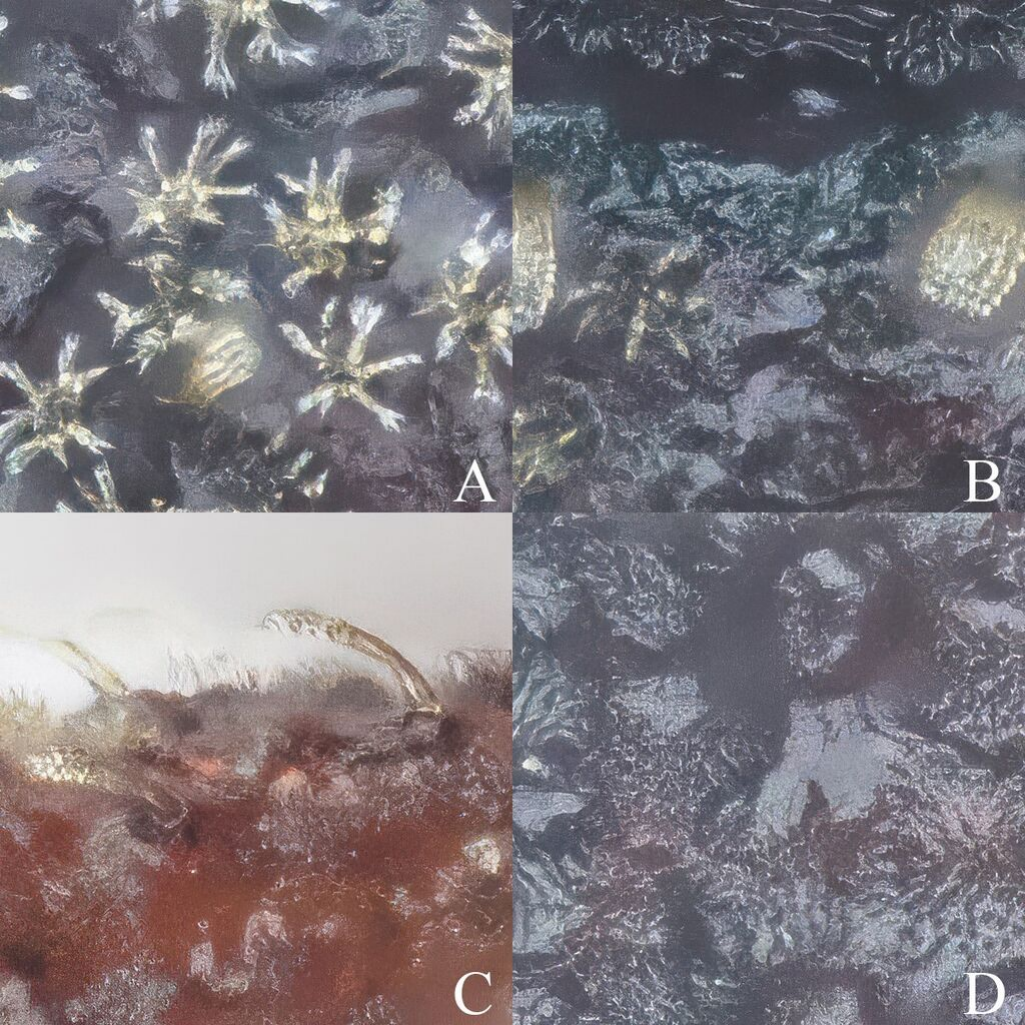
Microfeatures of *Trachyphloeosomarutiani* sp. nov., female. **A** Specialised star-shaped yellowish setae on pronotum; **B** Scutellar shield; **C** Flocculent microhair on legs; **D** Flocculent microhair on elytron.

Pronotum (Fig. 2A and Fig. 5A) 1.04× wider than long, widest at mid-length, hexagonal, constricted behind anterior margin. Anterior margin distinctly narrower than posterior margin. Disc finely rugose and punctate, intensified into a large, moderately wide longitudinal medial furrow along entire length, with ill-defined margins. Except for medial furrow, entire pronotum covered by star-shaped yellowish short setae, with interval erect yellowish setae at outer margins.

Elytra (Fig. 2A and Fig. 5B, D) elongated oval, 1.29× longer than wide, widest at mid-length, sides regularly rounded. Striae coarsely punctate, twice as wide as interstriae, each punctate circular. Interstriae densely covered by weak specialised star-shaped, yellowish, short setae; with yellowish erect setae on only odd interstriae, gradually more developed from base to apex and from interstria 1 to interstria 7, all setae not narrower than interstriae on declivity. Scutellar shield small, trapezoidal, margin indistinct (Fig. 5B).

Protibiae (Fig. 4A) coriaceous, long and slender, covered by weak, specialised, star-shaped, yellowish, short setae, 6.25× longer than width at mid-length, apically obliquely subtruncate, with dense fringe of fine yellowish setae, with robust yellowish mucro. Tarsi short and thick, claws free.

**Figure 6.**
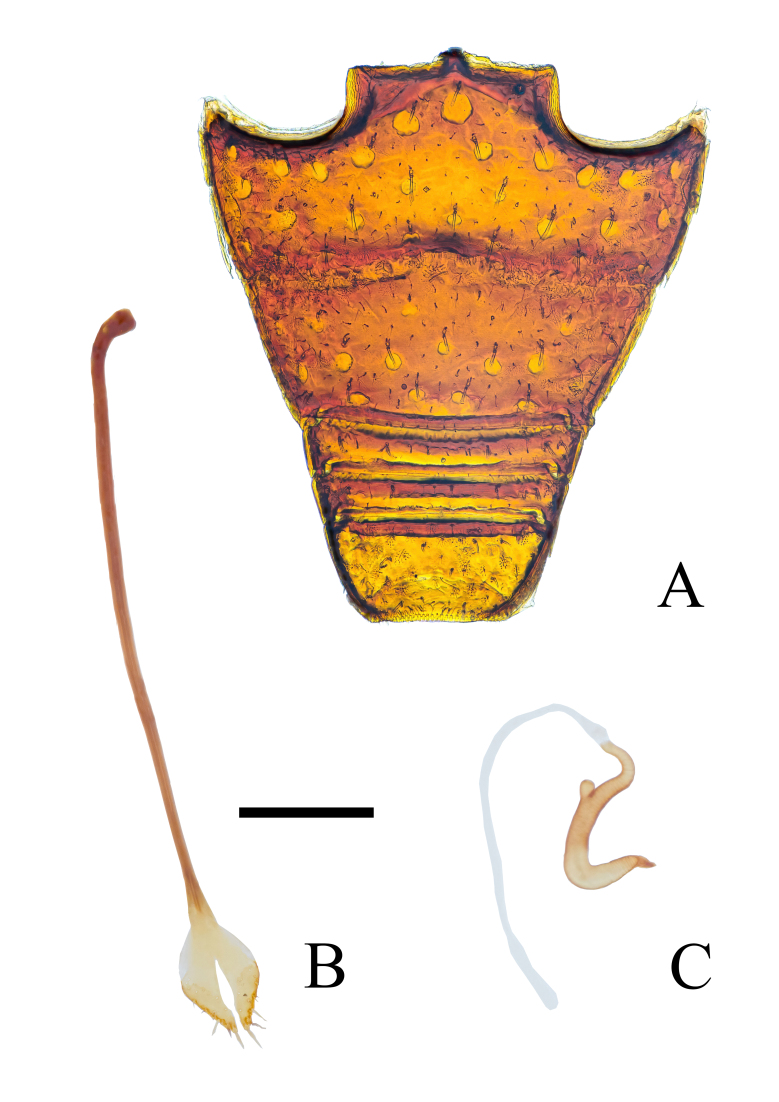
Abdominal ventrites and female genitalia of *Trachyphloeosomarutiani* sp. nov. **A** Abdominal ventrites 1 to 5; **B** Sternite VIII; **C** Spermatheca. Scale Bar = 0.2 mm.

Abdomen (Fig. 6A) 1.13× longer than wide. Ventrite 1+2 sparsely roughly punctate, ventrite 1 slightly longer than 2, ventrite 2 visibly longer than 3+4, ventrite 5 covered by specialised, star-shaped, yellowish, short setae Suture 1 fine, slightly sinuous, surrounded by specialised, star-shaped, yellowish, short setae, other sutures straight, deep. Intercoxal process of abdominal ventrite 1 flat.

Female genitalia (Fig. 6B and C): Sternite VIII with long, slender apodeme, about 4× as long as plate; plate rhombic, with distinct, slender, medial longitudinal emargination extending along distal 2/3 of plate. Spermatheca with long and irregularly curved cornu; corpus slender, indistinct; ramus small, slightly longer than wide; collum distinctly irregularly curved, apex truncated, exactly reaching extended line of corpus.

**Male.** Unknown.

#### Diagnosis

By having a longitudinal median furrow along the entire length of the pronotum, *Trachyphloeosomarutiani* sp. nov. is similar to *T.ales* Borovec & Anderson 2022. They can be distinguished by *T.rutiani* sp. nov. having erect setae on elytra on only odd interstriae, the shape of the scrobes (scrobes more straight and sharp in *T.ales*, also opened at posteroventral margin; gradually enlarged posteriad into a separated reniform groove before eyes, opened at posteroventral margin in *T.rutiani* sp. nov.), ridges behind eyes (*T.ales* has ridges behind eyes at the same level as the eyes; *T.rutiani* sp. nov. much higher), shape of the plate of sternite VIII (*T.ales* with entire plate of sternite VIII; *T.rutiani* sp. nov. with distinct, slender, longitudinal emargination extending along distal 2/3 of plate), shape of spermatheca (*T.ales* has ramus and collum not developed; *T.rutiani* sp. nov. has ramus small, collum distinctly irregularly curved).

By having elevated ridges behind the eyes and slender and long protibiae, *T.rutiani* is similar to *T.jirka* Ren, Borovec & Zhang 2020; they can be distinguished by *T.rutiani* sp. nov. having a longitudinal median furrow along the entire length of the pronotum, having erect setae on elytra on only odd interstriae, by the shape of the plate of sternite VIII (*T.jirka* with entire plate of sternite VIII; *T.rutiani* sp. nov. with distinct, slender, longitudinal emargination extending along distal 2/3 of plate), shape of spermatheca (*T.jirka* has ramus not developed, collum very small, hump-shaped; *T.rutiani* sp. nov. has ramus small, collum distinctly irregularly curved).

By the shape of the female terminalia, *T.rutiani* sp. nov. is similar to *T.martin* Ren, Borovec & Zhang 2020; they can be distinguished by *T.rutiani* sp. nov. having a longitudinal median furrow along the entire length of the pronotum (more like *T.ales*), elevated ridges behind the eyes (more like *T.jirka*), slender proleg (more like *T.jirka*), longer elytra, strongly convex postgena (more like *T.jirka*; much more flat in *T.martin*), the shape of the plate of sternite VIII (*T.martin* with longitudinal emargination extending along distal 1/2 of plate and plate rounder), different shape of the spermatheca, long and irregularly curved cornu, ramus moderately longer than wide, collum long and irregularly curved.

*T.rutiani* sp. nov. is quite similar to *T.roelofsi* Sharp, 1896. A differential diagnosis is presented in Table 1.

#### Etymology

The species is named after one of the collectors, Ru-tian Ye [叶如天], younger brother of the third author, who participated in the fieldwork. The name is a noun in the genitive case.

#### Distribution

**Figure 7.**
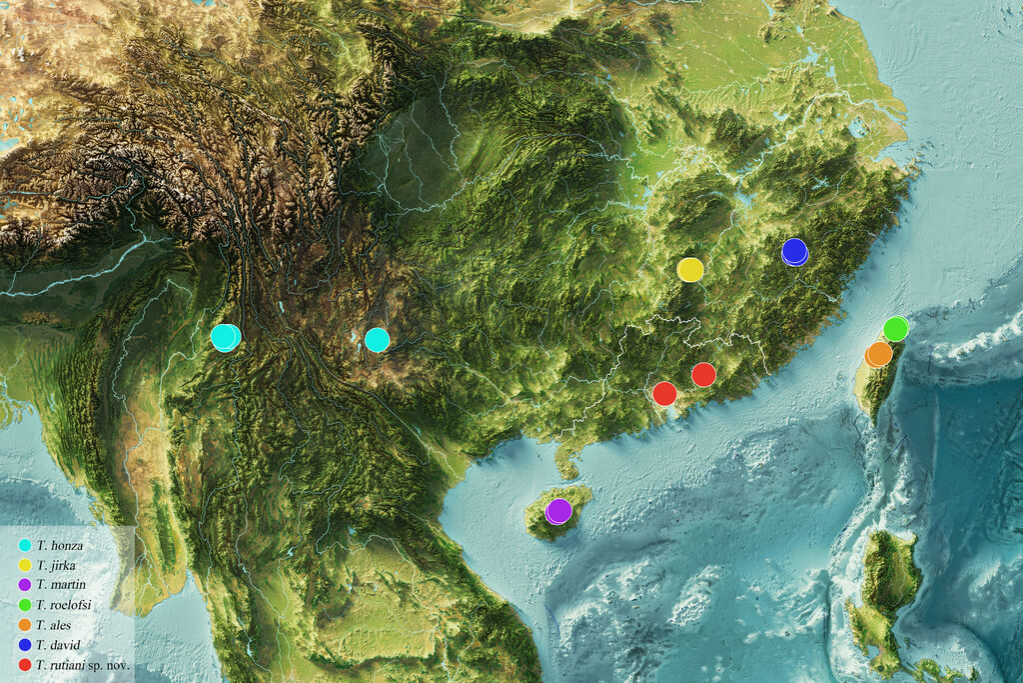
Geographical distribution of *Trachyphloeosoma* spp. in China, the outline of Guangdong Province has been marked with white line on the map.

China (Guangdong) (Fig. 7).

#### Biology

**Figure 8.**
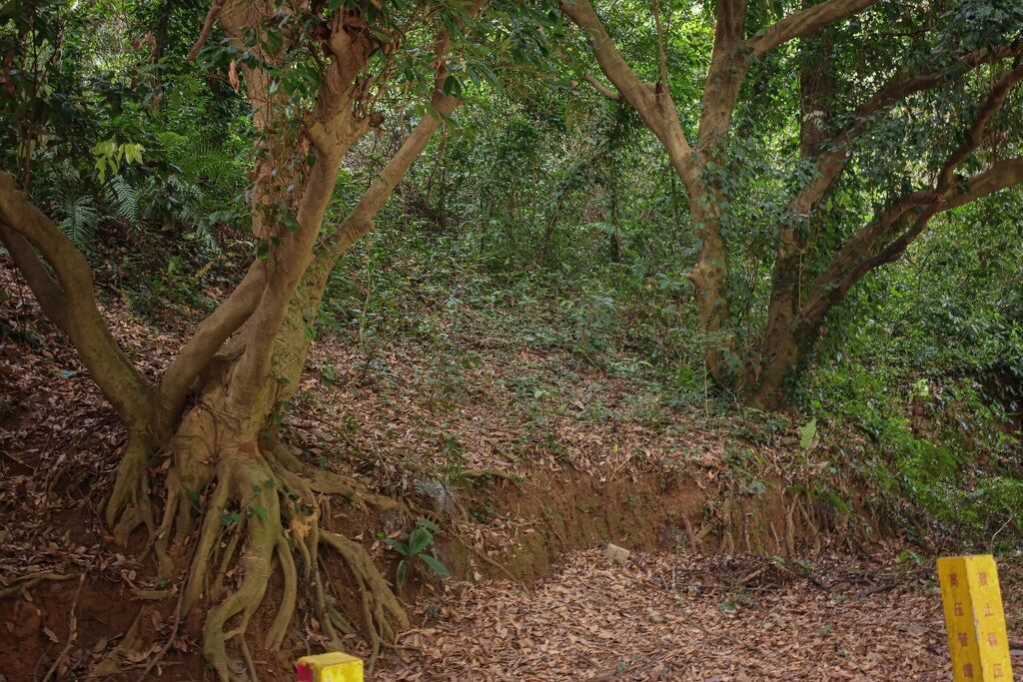
Habitat of *Trachyphloeosomarutiani* sp. nov. in leaf litter.

All specimens were collected in the deciduous layer of a mixed secondary broad-leaved forest and bamboo forest, mainly composed of *Ficusconcinna* (Moraceae), *Litchichinensis* (Sapindaceae), *Castanopsishystrix* (Fagaceae) and *Blechnopsisorientalis* (Blechnaceae) (Fig. 8).

#### Notes

Morphological characters of the new species are similar to *T.roelofsi* (Japan; Taiwan, China), *T.ales* (Taiwan, China), *T.jirka* (Vietnam; Jiangxi, China) and *T.martin* (Hainan, China) and their geographical distributions are close to each other, many features on the head can only be observed after clearing out the earth-like encrustation. The *Trachyphloeosoma*, as a wingless terricolous beetle genus north to Japan in Asia, may still have enormous potential in terms of species diversity in the further north part of China.

## Supplementary Material

XML Treatment for Trachyphloeosoma rutiani

## Figures and Tables

**Table 1.** Differential diagnosis between *T.rutiani* and *T.roelofsi*.

	*T.rutiani* sp. nov.	*T.roelofsi*
Body length	2.26–2.41 mm	2.00–2.30 mm
Head	with groove on frons divided into several sculptures and elevated ridges behind eyes	coriaceous, rugose, with dense oblong punctures, these on head not forming sulci
Pronotum disc	with longitudinal median furrow along the entire length (similar to *T.ales*)	regularly domed
Scutellar shield	small, trapezoidal, margin indistinct	invisible
Interstriae	with setae in odd interstriae, all interstriae finely punctate	with setae in odd interstriae, all interstriae nearly smooth
Protibiae	long and slender (6.25x longer than wide at mid-length, similar to *T.jirka*)	short (4.8-5.3x longer than wide at mid-length)（Ren et al. 2020）
Sternite VIII	Nearly same, also similar to *T.advena* and *T.buruana*.	
Spermatheca	Although spermatheca is variable in shape, *T.rutiani* do have a longer and more irregularly curved cornu, ramus moderately longer than wide, collum long and irregularly curved (more similar to *T.martin*).	
